# Flavonoids as key players in cold tolerance: molecular insights and applications in horticultural crops

**DOI:** 10.1093/hr/uhae366

**Published:** 2025-01-02

**Authors:** Jiaxin Li, Qinhan Yu, Chang Liu, Ningbo Zhang, Weirong Xu

**Affiliations:** College of Enology & Horticulture, Ningxia University, No.498 Helanshan West Street, Xixia District, Yinchuan, Ningxia 750021, China; School of Life Science, Ningxia University, No.498 Helanshan West Street, Xixia District, Yinchuan, Ningxia 750021, China; School of Life Science, Ningxia University, No.498 Helanshan West Street, Xixia District, Yinchuan, Ningxia 750021, China; College of Enology & Horticulture, Ningxia University, No.498 Helanshan West Street, Xixia District, Yinchuan, Ningxia 750021, China; Engineering Research Center of Grape and Wine, Ministry of Education, Ningxia University, No.498 Helanshan West Street, Xixia District, Yinchuan, Ningxia 750021, China; Key Laboratory of Modern Molecular Breeding for Dominant and Special Crops in Ningxia, No.498 Helanshan West Street, Xixia District, Yinchuan 750021, China; College of Enology & Horticulture, Ningxia University, No.498 Helanshan West Street, Xixia District, Yinchuan, Ningxia 750021, China; School of Life Science, Ningxia University, No.498 Helanshan West Street, Xixia District, Yinchuan, Ningxia 750021, China; Engineering Research Center of Grape and Wine, Ministry of Education, Ningxia University, No.498 Helanshan West Street, Xixia District, Yinchuan, Ningxia 750021, China; Key Laboratory of Modern Molecular Breeding for Dominant and Special Crops in Ningxia, No.498 Helanshan West Street, Xixia District, Yinchuan 750021, China; State Key Laboratory of Efficient Production of Forest Resources, No.498 Helanshan West Street, Xixia District, Yinchuan 750021, China

## Abstract

Cold stress profoundly affects the growth, development, and productivity of horticultural crops. Among the diverse strategies plants employ to mitigate the adverse effects of cold stress, flavonoids have emerged as pivotal components in enhancing plant resilience. This review was written to systematically highlight the critical role of flavonoids in plant cold tolerance, aiming to address the increasing need for sustainable horticultural practices under climate stress. We provide a comprehensive overview of the role of flavonoids in the cold tolerance of horticultural crops, emphasizing their biosynthesis pathways, molecular mechanisms, and regulatory aspects under cold stress conditions. We discuss how flavonoids act as antioxidants, scavenging reactive oxygen species (ROS) generated during cold stress, and how they regulate gene expression by modulating stress-responsive genes and pathways. Additionally, we explore the application of flavonoids in enhancing cold tolerance through genetic engineering and breeding strategies, offering insights into practical interventions for improving crop resilience. Despite significant advances, a research gap remains in understanding the precise molecular mechanisms by which specific flavonoids confer cold resistance, especially across different crop species. By addressing current knowledge gaps, proposing future research directions and highlighting implications for sustainable horticulture, we aim to advance strategies to enhance cold tolerance in horticultural crops.

## Introduction

Horticultural crops often face various environmental stresses, with cold stress being a significant factor that limits their growth, development, and productivity. Cold stress encompasses both chilling temperatures (0°C–18°C) and freezing conditions (<0°C), posing substantial challenges to crop cultivation [1]. This stress disrupts plant physiology, impairs photosynthesis, and compromises root development, leading to reduced fruit yield and quality [2]. Economically, cold stress results in substantial financial losses for farmers and the horticultural industry, manifesting as reduced yields, delayed harvests, decreased product quality, and total crop failures [3, 4]. In temperate regions, frost events account for >30% of weather-related insured crop losses, significantly impacting smallholdings and communities reliant on stable harvests [5]. Therefore, developing cold-tolerant horticultural crops is crucial for ensuring stable production and economic resilience [6]. Despite the recognition of these challenges, current research efforts remain insufficient. There is an urgent need for a multidisciplinary approach that emphasizes horticultural practices alongside advancements in biotechnology to effectively address cold stress in horticultural crops.

Plants have evolved complex mechanisms to endure cold stress [7], with flavonoids emerging as key components in their stress responses [8]. Flavonoids, a class of secondary metabolites, enhance plant resilience to various stressors, including cold stress [9, 10]. They mitigate oxidative stress by acting as antioxidants, scavenging reactive oxygen species (ROS) generated under cold conditions [11, 12]. The accumulation of flavonoids in response to cold stress is observed in various plant species, indicating a common adaptive mechanism [13]. Increased flavonoid levels are associated with enhanced resistance to abiotic stresses, including cold stress [14]. Furthermore, flavonoids regulate gene expression in response to cold stress, modulating stress-responsive genes and pathways (Table 1). Studies have identified key structural gene families involved in flavonoid biosynthesis, emphasizing their genetic mechanisms and impact on stress responses [22]. These findings highlight the potential for genetic and metabolic engineering to enhance cold tolerance in horticultural crops.

**Table 1:** Key pathways involved in cold stress response and flavonoid metabolism in plants

**Pathway name**	**Role in cold stress response or flavonoid metabolism**	**References**
Phenylpropanoid pathway	Initiates flavonoid biosynthesis; contributes to ROS scavenging under cold stress by producing key intermediates like cinnamic acid.	[14, 15]
CBF-COR pathway	Cold-responsive (*CBF*) genes activate *COR* genes, increasing anthocyanin levels that support antioxidative responses in cold tolerance.	[16]
MYB transcription factor pathway	Regulates expression of flavonoid biosynthetic genes (e.g. *CHS*, *DFR*) under cold stress, influencing anthocyanin and proanthocyanidin accumulation.	[17]
Jasmonic acid (JA) signaling pathway	Enhances flavonoid accumulation under stress, supporting membrane stability and oxidative defense mechanisms.	[18]
Abscisic acid (ABA) signaling pathway	Induces flavonoid biosynthesis in response to cold, strengthening ROS scavenging capabilities and cell membrane protection.	[19]
MAPK signaling pathway	Modulates MYB transcription factors and interacts with flavonoid pathways to regulate ROS levels during cold stress.	[20]
Calcium signaling pathway	Activates cold-responsive genes via calcium flux, which enhances flavonoid biosynthesis and strengthens antioxidative responses.	[21]

In this review, we first summarize the key discoveries related to flavonoid biosynthesis pathways, molecular mechanisms, and regulatory aspects under cold stress conditions. We then discuss how flavonoids function as antioxidants, scavenging ROS generated during cold stress, and how they regulate gene expression by modulating stress-responsive genes and pathways. Furthermore, we explore the application of flavonoids in enhancing cold tolerance through genetic engineering and breeding strategies. Finally, we address current knowledge gaps, propose future research directions, and highlight the implications for sustainable horticulture, aiming to advance strategies for enhancing cold tolerance in horticultural crops.

## Flavonoid biosynthesis pathways

Flavonoid biosynthesis pathways are intricate processes regulated by key enzymes and genes, controlling the production of these essential secondary metabolites. In this section, we review the enzymes and genes involved in flavonoid biosynthesis, the regulation of flavonoid production under cold stress, and the cross-talk with other pathways, providing insights into the molecular mechanisms that control flavonoid biosynthesis in response to environmental cues.

## Key enzymes and genes involved

**Figure 1:**
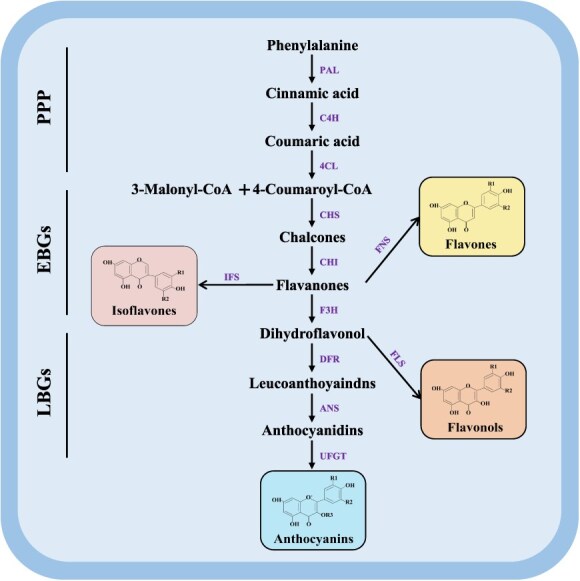
Biosynthetic pathways of flavonoids in plants. The diagram delineates the sequential enzymatic steps converting phenylalanine into key flavonoid compounds: isoflavones, flavones, flavonols, and anthocyanins. Beginning with the transformation of phenylalanine into cinnamic acid via phenylalanine ammonia-lyase (*PAL*), the pathway proceeds through cinnamate 4-hydroxylase (*C4H*) and 4-coumarate-CoA ligase (*4CL*) to produce 3-Malonyl-CoA and 4-Coumaroyl-CoA. Chalcone synthase (*CHS*) then catalyzes the formation of chalcones, which are isomerized by chalcone isomerase (*CHI*) to produce flavanones. Flavanones are diversified into different flavonoid classes: isoflavones via isoflavone synthase (*IFS*), flavones via flavone synthase (*FNS*), and further conversion into dihydroflavonols by flavanone 3-hydroxylase (*F3H*). Dihydroflavonols are converted to leucoanthocyanidins by dihydroflavonol 4-reductase (*DFR*), which are subsequently transformed into anthocyanidins by anthocyanidin synthase (*ANS*) and stabilized into anthocyanins by UDP-glucose: flavonoid 3-O-glucosyltransferase (*UFGT*). Alternatively, dihydroflavonols can be converted to flavonols by flavonol synthase (*FLS*). This pathway highlights the metabolic intricacies and enzymatic specificity required for flavonoid biosynthesis, crucial for plant coloration, UV protection, and interactions with pollinators. PPP, phenylpropanoid pathway; EBGs, flavonoid early biosynthetic genes; LBGs, flavonoid late biosynthetic genes.

Flavonoid biosynthesis involves a series of enzyme-catalyzed reactions essential for producing flavonoid compounds [23]. These enzymes include chalcone synthase (CHS), chalcone isomerase (CHI), flavanone 3-hydroxylase (F3H), flavonol synthase (FLS), leucoanthocyanidin dioxygenase (LDOX), anthocyanidin synthase (ANS), flavone synthase II (FNSII), flavanone 2-hydroxylase (F2H), flavonoid 3′-hydroxylase (F3’H), and flavonoid 3′,5′-hydroxylase (F3’5’H). These enzymes catalyze the conversion of precursor molecules into various flavonoid compounds, contributing to the diverse array of flavonoids found in plants (Table 2 and Fig. 1). Understanding the specific roles and regulation of these enzymes offers valuable insights into the genetic manipulation of flavonoid biosynthesis, which could enhance flavonoid production and improve the stress resilience of horticultural crops.

**Table 2:** Summary of the key enzymes involved in the biosynthesis of flavonoids and their specific functions within the pathway, along with supporting references

**Enzyme**	**Function**	**References**
Phenylalanine ammonia-lyase (PAL)	Converts phenylalanine to cinnamic acid, initiating the phenylpropanoid pathway.	[24]
Cinnamate 4-hydroxylase (C4H)	Hydroxylates cinnamic acid to form p-coumaric acid.	[24]
4-Coumarate-CoA ligase (4CL)	Activates p-coumaric acid to p-coumaroyl-CoA.	[24]
Chalcone synthase (CHS)	Catalyzes the formation of chalcones from p-coumaroyl-CoA, the first step in flavonoid biosynthesis.	[25]
Chalcone isomerase (CHI)	Isomerizes chalcones to form flavanones.	[25]
Flavanone 3-hydroxylase (F3H)	Hydroxylates flavanones to form dihydroflavonols.	[26]
Dihydroflavonol 4-reductase (DFR)	Reduces dihydroflavonols to leucoanthocyanidins.	[26]
Anthocyanidin synthase (ANS)	Converts leucoanthocyanidins to anthocyanidins.	[26]
Flavonol synthase (FLS)	Converts dihydroflavonols to flavonols.	[26]
UDP-glucose 3-O-glucosyltransferase (UFGT)	Glycosylates anthocyanidins to stabilize anthocyanins.	[25]
Isoflavone synthase (IFS)	Converts flavanones to isoflavones.	[27]
Flavone synthase (FNS)	Converts flavanones to flavones.	[28]

The biosynthesis of flavonoids begins in the phenylpropanoid pathway, where the amino acid phenylalanine is converted into cinnamic acid by phenylalanine ammonia-lyase (*PAL*) [29]. This marks the entry into a complex network of enzymatic reactions leading to the production of various flavonoid skeletons [30]. Subsequent steps involve hydroxylations, methylations, glycosylations, and acylations, catalyzed by enzymes such as CHS, CHI, and F3’H. These reactions culminate in the formation of diverse flavonoid classes, including flavones, flavonols, isoflavones, and anthocyanins [31–33]. This intricate pathway highlights the biochemical versatility of plants and provides numerous targets for metabolic engineering. Manipulating specific enzymes within this pathway can enhance the production of certain flavonoids, bolstering the plant’s ability to withstand various environmental stresses.

## Genetic regulation of flavonoid synthesis

The expression of genes encoding enzymes involved in flavonoid biosynthesis is tightly controlled by a network of transcription factors that bind to the promoters of these genes, affecting flavonoid synthesis [34, 35]. These transcription factors respond to both internal developmental signals and external environmental cues, orchestrating a finely tuned regulatory system. Integrating transcriptome and metabolome data has provided new insights into the biosynthesis and regulation mechanisms of flavonoids in various plant species [36–40]. For instance, flavonoid biosynthesis is regulated by various transcription factors, such as R2R3-MYB proteins [41, 42]. Understanding these networks opens possibilities for targeted genetic modifications to enhance specific flavonoid pathways, thereby improving plant resilience and nutritional value.

Recent studies have revealed the role of specific MYB transcription factors in regulating flavonoid biosynthesis under cold stress, suggesting a genetic basis for flavonoid level regulation in response to cold. For example, in apple (*Malus domestica*), MYB transcription factors such as *MdMYB308L*, *MdbHLH33*, *MdMYB12*, *MdMYB22*, and *MdMYB114* play crucial roles in cold tolerance and anthocyanin accumulation. MdMYB308L interacts with MdbHLH33 to enhance binding to the *MdCBF2* and *MdDFR* promoters, while the RING E3 ubiquitin ligase MdMIEL1 promotes the degradation of MdMYB308L, regulating cold tolerance and anthocyanin accumulation [43]. The promoters of *MdMYB12*, *MdMYB22*, and *MdMYB114* contain CBF/DREB response elements activated under cold conditions, closely relating their expression to anthocyanin accumulation [44]. These insights into MYB-mediated regulation of flavonoid biosynthesis under cold stress elucidate the molecular mechanisms of cold tolerance and provide potential targets for genetic manipulation to enhance cold hardiness and crop quality.

Cold stress significantly induces the apple R2R3-MYB transcription factor *MdMYB23*, which activates *MdCBF1* and *MdCBF2* expression and interacts with the *MdANR* promoter, enhancing proanthocyanidin accumulation and ROS scavenging in apple and *Arabidopsis thaliana* [45]. In apple, MYB transcription factors involved in proanthocyanidin synthesis can be categorized into TT2 and PA1 types. *MdMYBPA1*, a PA1-type MYB transcription factor, shifts the flavonoid biosynthesis pathway from proanthocyanidin to anthocyanin biosynthesis under cold stress, with coexpression of *MdMYBPA1* and *MdbHLH33* producing more anthocyanins. *MdbHLH33* binds to the low-temperature-responsive element of the *MdMYBPA1* promoter, activating its function [46]. In blood orange (*Citrus sinensis* cv Tarocco), a retrotransposon in the *Ruby1* promoter, encoding an R2R3-MYB transcription factor, regulates cold-induced anthocyanin accumulation. ETHYLENE RESPONSE FACTORS (CsERF054 and CsERF061) activate *CsRuby1* by binding to a DRE/CRT element within the retrotransposon [47]. The transcriptional activity of MITOGEN-ACTIVATED PROTEIN KINASE3 (*FvMAPK3*) is induced to phosphorylate *FvMYB10* and *FvCHS1* under cold stress, enhancing their proteasome-mediated degradation process and negatively regulating anthocyanin accumulation, thereby increasing the cold sensitivity of plants [48]. Under cold stress, the RING-type E3 ubiquitin ligase CsMIEL1 from the tea plant (*Camellia sinensis*) regulates the excessive accumulation of anthocyanins by binding to CsMYB90 and eliminating the activation of downstream genes such as *CsUF3GT*, *CsDFR*, and *CsANS* [49]. These studies highlight the intricate regulatory networks involving MYB transcription factors and other interacting proteins that modulate flavonoid biosynthesis under cold stress. Understanding these mechanisms enables the development of strategies to enhance cold tolerance and flavonoid content in various horticultural crops.

## Specific flavonoids involved in plant stress responses

Among the various flavonoids produced by plants, certain compounds are specifically associated with responses to abiotic stress, including cold stress. For instance, anthocyanins accumulate in plant tissues exposed to cold stress, conferring protection against cold-induced photoinhibition and oxidative stress [50]. Flavonols, such as quercetin and kaempferol, play critical roles in scavenging ROS and stabilizing cellular membranes under stress conditions [51]. In bananas (*Musa* spp. ABB Pisang Awak), the *MaC2H2-like* transcription factor activates genes related to flavonoid synthesis, increasing flavonoid content and reducing cold damage [52]. In transgenic woodland strawberry (*Fragaria vesca*), cold stress is associated with anthocyanin biosynthesis and enhanced cold tolerance [53]. Red mango (*Mangifera indica* L.) varieties with high anthocyanin content show decreased decay rates and increased cold tolerance [54]. Overexpression of *AaLAR1* in kiwifruit (*Actinidia arguta*) improves cold tolerance by promoting proanthocyanidin biosynthesis and activating the antioxidant system [55]. These examples illustrate the diverse roles of different flavonoids in enhancing cold stress tolerance across various plant species.

Cold stress stimulates the conversion of dihydroflavonols to anthocyanins in blood oranges (*Citrus sinensis* L. Osbeck), promoting anthocyanin accumulation [56]. In grape (*Vitis vinifera* L.) berries, cold stress increases the expression of anthocyanin biosynthesis genes (*CHS3*, *F3H1*, and *UFGT*) and anthocyanin content [57]. In *Tetrastigma hemsleyanum* Diels et Gilg, cold stress induces the expression of flavonoid biosynthetic genes (*PAL*, *C4H*, *4CL*, *CHS*, *CHI*, *F3H*, *ANR*, *FLS*, and *LAR*), increasing total flavonoid content and antioxidant capacity [58]. Transgenic *A. thaliana* shows increased synthesis of flavonoids, including flavonols, anthocyanins, and flavanones, under cold stress, improving cold tolerance [59]. The differential accumulation of flavonoids in response to cold stress highlights the adaptive significance of flavonoid biosynthesis in enhancing plant resilience to unfavorable environmental conditions.

## Crosstalk with other metabolic pathways

Flavonoid biosynthetic pathways interact with various other metabolic pathways, forming a complex network that regulates plant responses to environmental stimuli [60–62]. Flavonoid biosynthesis is linked to pathways involved in phenylpropanoid metabolism, hormone signaling, and stress responses. For example, flavonoids interact with phytohormone signaling pathways, such as jasmonate and abscisic acid, to modulate plant defense mechanisms under cold stress [63, 64]. Additionally, flavonoids participate in signaling cascades that regulate gene expression, metabolic processes, and stress responses [65–67]. This intricate interplay suggests that enhancing flavonoid biosynthesis could have cascading effects on multiple stress response pathways, providing a comprehensive defense strategy.

## Molecular mechanisms of flavonoid action in cold tolerance

### Antioxidant properties of flavonoids

Flavonoids act as potent antioxidants, conferring cold resistance in horticultural crops. Cold stress leads to an overproduction of ROS such as superoxide anions, hydrogen peroxide, and hydroxyl radicals, causing oxidative damage to lipids, proteins, and nucleic acids, and disrupting cellular function [68, 69]. Flavonoids mitigate oxidative stress by scavenging ROS, thus protecting cells from damage [70–72]. Their antioxidant activity is largely attributed to the presence of hydroxyl groups [73, 74]. Studies have shown that flavonoid accumulation in plants correlates with enhanced antioxidative capacity and increased cold tolerance. For instance, the flavonoid biosynthetic pathway and ROS-scavenging capacity are rapidly enhanced during early cold stress in winter rapeseed (*Brassica napus* L.), significantly improving cold tolerance [75].

Recent advances in high-throughput metabolomics and transcriptomics have profoundly enhanced our understanding of flavonoid functions in cold tolerance across diverse horticultural crops. These approaches allow for comprehensive profiling of gene expression and metabolites, offering valuable insights into the cold-induced modulation of flavonoid pathways. For instance, in *Pyrus hopeiensis*, Li *et al.* [39] identified key cold-responsive genes and transcription factors (e.g. bHLH, MYB) that regulate flavonoid biosynthesis, providing a genetic basis for breeding cold-resistant pears. In *Medicago sativa*, flavonoid pathway modulation under combined cold and saline–alkali stresses highlighted *MsMYB12*’s role in flavonol accumulation, supporting multistress resilience [76]. Sun *et al.* [77] showed that cold-tolerant *A. arguta* genotypes enhance ROS scavenging via upregulation of CHI and anthocyanin pathway genes. Similarly, multiomics analyses in *Solanum lycopersicum* linked MYB-regulated flavonoid biosynthetic genes to secondary metabolism responses under cold storage [40]. Studies on *Fagopyrum tataricum* [78] and *Prunus persica* [79] further identified genotype-specific flavonoid pathway activations that bolster cold tolerance through enhanced phenylpropanoid biosynthesis. Collectively, these integrative omics studies underscore flavonoids’ central role in cold adaptation and offer precise molecular targets for breeding frost-resilient horticultural crops.

Cold stress induces the expression of genes such as *AtCHS1*, *AtCHI4*, *AtF3H1*, and *AtDFR2* in transgenic *A. thaliana*, leading to increased anthocyanin content, inhibition of ROS, induced stomatal closure, and reduced electrolyte loss, enhancing cold tolerance [80]. In apple, *MdNAC104* stimulates anthocyanin accumulation by upregulating synthesis-related genes, inhibiting excessive ROS accumulation, and improving cold resistance [15]. In tomato (*S. lycopersicum* L.), the bHLH transcription factor regulates anthocyanin biosynthesis, enhancing cold tolerance by upregulating anthocyanin biosynthesis genes [81]. In tea plants, the glycosyltransferase *CsUGT78A14*, strongly induced by cold stress, enhances antioxidant capacity by scavenging ROS [82]. Cold stress also induces higher levels of catechins in tea plants, improving their resistance to cold stress [83]. These examples illustrate the multifaceted roles of genes and transcription factors in mediating flavonoid biosynthesis and enhancing cold tolerance.

### Role of flavonoids in modulating gene expression under cold stress

**Figure 2:**
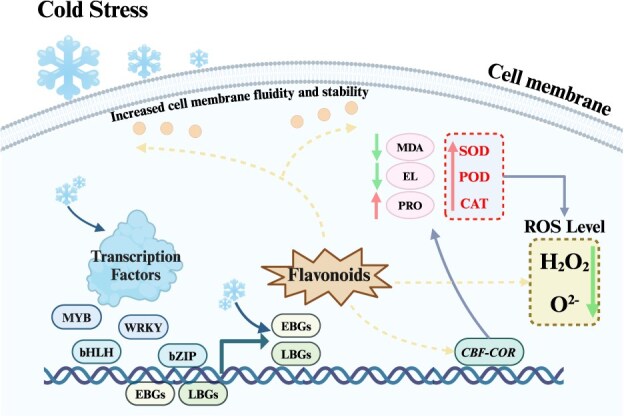
Regulation mechanism of flavonoids under cold stress. The diagram depicts how cold stress induces the accumulation of flavonoids in plants through the activation of various transcription factors (MYB, WRKY, bHLH, bZIP), which either regulate the expression of flavonoid synthesis genes or directly induce their expression. Flavonoids, acting as potent antioxidants, play a critical role in scavenging ROS such as hydrogen peroxide (H_2_O_2_) and superoxide anion (O^2−^), thereby reducing oxidative damage. These compounds also function as signaling molecules that modulate the expression of cold-responsive (*CBF*-*COR*) genes. Furthermore, flavonoids enhance cell membrane fluidity and stability, maintaining cellular homeostasis under cold stress. This multifaceted response involves a reduction in malondialdehyde (MDA) and electrolyte leakage (EL), and an increase in proline (PRO) levels, mediated by antioxidant enzymes such as SOD, peroxidase (POD), and catalase (CAT). This comprehensive regulatory network highlights the crucial role of flavonoids in enhancing plant tolerance to cold stress. EBGs, flavonoid early biosynthetic genes; LBGs, flavonoid late biosynthetic genes; *CBF*, C-repeat binding transcription factor; *COR*, cold-responsive gene. Figure created with Biorender (https://www.biorender.com/).

Flavonoids regulate the expression of genes involved in the cold stress response at multiple levels, from transcriptional activation of cold-responsive (*COR*) genes to posttranslational modifications of proteins involved in stress signaling pathways (Fig. 2). Flavonoids interact with transcription factors such as MYB, bHLH, WRKY, and bZIP to activate cold-responsive genes [22, 84]. These genes encode proteins like chaperones, antifreeze proteins, and enzymes for osmoprotectant biosynthesis, which protect cells from cold-induced damage [85, 86]. Flavonoids may act as signaling molecules themselves or modulate the activity of other signaling components [87].

Under cold stress, WRKY transcription factors induce the expression of genes such as *AtCOR413*, *AtCOR15B*, *AtCBF1*, and *AtCBF2*, promoting anthocyanin accumulation and improving cold tolerance [80]. The bZIP transcription factor *SlAREB1* enhances anthocyanin biosynthesis under cold stress via the abscisic acid (ABA) signaling pathway [88]. Overexpression of the bZIP transcription factor *AtHY5* in *Arabidopsis* improves flavonol accumulation under cold stress [89]. The bHLH transcription factor *MdbHLH3* activates anthocyanin biosynthetic genes in apple, and its phosphorylation under cold stress enhances its binding ability and transcriptional activity [90]. Moreover, *MdbHLH3* can bind directly to the low-temperature-responsive *cis*-element in the promoter of *MdBBX20*, thereby regulating its expression. *MdBBX20* also binds to the promoters of *MdMYB1*, *MdDFR*, and *MdANS*, facilitating the biosynthesis of anthocyanins [91]. Additionally, the expression levels of *MdCBF2*, *MdCOR15A-1*, and *MdCOR15A-2* in transgenic apples increased, enhancing cold tolerance and adaptability to cold environments [65].

Building on these insights into transcription factor-mediated regulation, recent studies have further highlighted the multifaceted role of flavonoids in cold stress tolerance across plant species. Flavonoids, through a sophisticated regulatory network, bolster cold tolerance by activating metabolic and transcriptional pathways linked to cryoprotection, osmoprotection, and antioxidative defense [92, 93]. For example, in Chrysanthemum [92] and cotton [93], cold-induced flavonoid accumulation correlates with significant gene expression shifts that enhance resilience to stress. Similarly, in sand rice and lettuce, adaptive activation of flavonoid biosynthesis pathways under cold conditions underpins effective low-temperature responses [94, 95]. Conversely, in rice, specific regulatory genes negatively modulate flavonoid biosynthesis under cold stress, underscoring a nuanced, species-specific regulation of these pathways [22]. Further studies in *Pyrus hopeiensis* and *Arabidopsis* reveal that flavonoids are integral to antioxidative systems, protecting cellular integrity against cold-induced oxidative stress [96, 97]. Additionally, research on tomato suggests that external application of seaweed extracts can influence flavonoid pathways to improve cold tolerance, demonstrating the potential for external modulation of these protective mechanisms [98]. Collectively, these findings elucidate the intricate role of flavonoid metabolism in cold stress adaptation, adding depth to our understanding of stress resilience at both transcriptional and metabolic levels.

### Membrane stabilization

Flavonoids modulate the physical properties of cellular membranes, as illustrated in Fig. 2. Flavonoids help stabilize cellular membranes, crucial for plant survival under cold stress [99, 100]. Cold stress alters membrane lipid composition, increasing rigidity and decreasing permeability [101, 102]. Flavonoids stabilize cell membranes by adjusting fluidity and permeability [103]. For example, cold stress promotes anthocyanin accumulation, upregulating structural genes and transcription factors that enhance grape leaf tolerance to cold stress [104].

Administering exogenous phenylalanine enhances flavonoid levels, alleviates electrolyte leakage in cellular membranes, and reduces malondialdehyde content, thereby maintaining membrane stability [105]. Flavonoids can incorporate into membranes, interacting with phospholipids and proteins, helping maintain membrane structure and function during cold stress [106]. This stabilization is essential for preserving the activity of membrane-bound enzymes and transporters, ensuring proper ion and molecule flow for cellular homeostasis [107]. By incorporating into membranes and interacting with key components, flavonoids help preserve membrane integrity and function, vital for maintaining cellular homeostasis.

### Interaction with other signaling pathways

Flavonoids interact with various signaling pathways to help plants respond to cold stress. These interactions involve cross-talk between flavonoid biosynthesis pathways and other stress-responsive pathways, such as hormone signaling and defense responses [20, 108]. Flavonoids serve as signaling molecules that integrate environmental cues and regulate downstream pathways to enhance cold tolerance [88, 89]. The intricate network of interactions between flavonoids and signaling molecules underscores their significance in mediating plant adaptation to cold stress.

### Cross-talk with hormone signaling pathways

**Figure 3:**
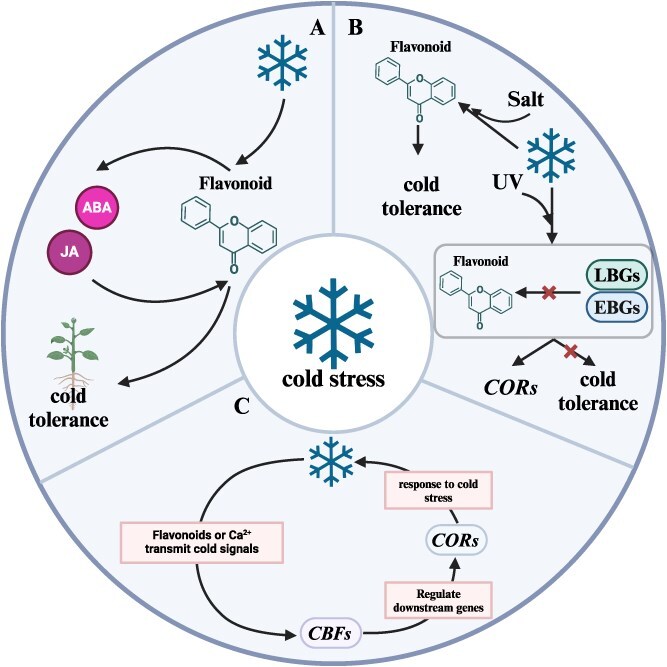
Crosstalk of flavonoids with other signaling pathways under cold stress. This diagram illustrates the complex interactions between flavonoids and various signaling pathways that enhance plant cold resistance. **A.** Under cold stress, the synthesis of flavonoids is promoted, which in turn enhances the production of ABA and JA. There exists a reciprocal relationship where ABA and JA also promote flavonoid synthesis, creating a positive feedback loop that enhances plant cold resistance. **B.** The combined effects of UV stress, salt stress, and cold stress regulate the content of flavonoids, which plays a critical role in modulating plant chilling resistance. These environmental stresses synergistically influence flavonoid pathways, thereby impacting the plant’s ability to withstand cold conditions. **C.** Cold stress induces the production of flavonoids or the activation of calcium channels, which operate through the *CBF* (C-repeat binding factor) signaling pathway. This pathway regulates the expression of downstream cold-responsive (*COR*) genes, ultimately aiding the plant’s response to cold stress. This integrated model highlights the multifaceted roles of flavonoids in enhancing cold resistance through interactions with hormonal pathways (ABA and JA), response to multiple environmental stresses (UV and salt), and the regulation of stress-responsive gene expression via the *CBF*-*COR* signaling pathway. EBGs, flavonoid early biosynthetic genes; LBGs, flavonoid late biosynthetic genes; *CBFs*, C-repeat binding transcription factors; *CORs*, cold-responsive genes. Figure created with Biorender (https://www.biorender.com/).

Flavonoids enhance cold tolerance by modulating hormone signaling pathways, such as ABA and jasmonic acid (JA) (Fig. 3a). Cold stress increases flavonoid levels, which influence ABA and JA biosynthesis and signaling [96, 109]. ABA and JA also affect flavonoid synthesis and, consequently, the cold resistance of plants [18, 110]. The abscisic acid-insensitive 5 (ABI5) transcription factor of the ABA signaling pathway is crucial in this process. Exogenous ABA treatment under cold stress increases the transcription levels of *MaABI5-like* and flavonoid synthesis-related genes, with *MaABI5-like* directly interacting with the promoters of these genes to activate their expression [111]. Transient overexpression of *MaABI5-like* in banana fruits and ectopic expression in tomato plants enhanced cold tolerance and upregulated the transcription levels of flavonoid synthesis-related genes, indicating that *MaABI5-like* regulates ABA-induced cold tolerance by increasing flavonoid content.

Exogenous JA sprayed on *Jatropha curcas* leaves increases the activity of *PAL* and enhances flavonoid content, including kaempferol, apigenin, and vitexin. JA induction in *J. curcas* also boosts the antioxidant response to maintain cellular redox balance [112]. In rice (*Oryza sativa* subsp. japonica), knockout lines for Jacalin-related lectin (*OsJRL*) accumulate more ABA and JA under cold stress, leading to higher flavonoid accumulation, reduced ROS levels, and significantly enhanced cold tolerance [22]. These examples demonstrate the interplay between flavonoids and hormone-signaling pathways in enhancing cold tolerance, showing that the increase in flavonoid content through JA and ABA signaling not only boosts antioxidant defenses but also reduces oxidative damage under cold stress.

In addition to ABA and JA, other phytohormones, such as ethylene (ET), salicylic acid (SA), gibberellins (GAs), strigolactone (SL), and brassinosteroids (BRs), also play essential roles in modulating flavonoid-mediated responses to cold stress. These hormones interact with flavonoid biosynthesis pathways to activate various defense mechanisms, including regulating ROS levels, stabilizing cellular structures, and conserving energy under cold conditions. Table 3 provides a summary of these key phytohormones and their roles in cold stress regulation, highlighting their specific contributions to enhancing cold tolerance through interactions with flavonoid pathways.

**Table 3:** Summary of key phytohormones involved in flavonoid-mediated cold stress regulation and their specific roles

**Phytohormone**	**Role in cold stress response**	**References**
Abscisic acid (ABA)	ABA serves as a critical signal for cold tolerance, regulating the expression of stress-responsive genes and stabilizing cellular functions. It influences flavonoid biosynthesis, enhancing oxidative stress resistance.	[113, 114]
Ethylene (ET)	ET is involved in freezing tolerance, regulating ROS levels and modulating antioxidant defenses through the flavonoid pathway, improving stress resilience and cellular stability.	[115, 116]
Salicylic acid (SA)	SA enhances resistance to cold-induced oxidative stress by activating antioxidant pathways and coordinating with flavonoid biosynthesis to manage ROS and stabilize cell structures.	[117, 118]
Gibberellins (GAs)	GAs downregulate under cold stress, conserving energy by inhibiting growth. Their interaction with ABA and the flavonoid pathway contributes to maintaining stress resilience.	[119, 120]
Jasmonic acid (JA)	JA enhances cold tolerance by activating defense-related genes and supporting flavonoid biosynthesis, which mitigates oxidative stress and improves cellular integrity.	[96, 121]
Strigolactone (SL)	SLs enhance cold tolerance by activating CBF pathway genes and modulating transcription factors such as *WRKY41* and *HY5*. This regulation alleviates repression on *CBF* expression, boosts antioxidant activity, and facilitates protein degradation, collectively strengthening plant resilience to freezing stress.	[122–124]
Brassinosteroids (BRs)	BRs enhance chilling tolerance by promoting ABA biosynthesis through *NCED1* upregulation and activating CBF-dependent pathways under cold stress. These actions increase antioxidant enzyme activity and stabilize cell structures, effectively mitigating chilling injury symptoms and enhancing plant resilience in low-temperature conditions.	[125–127]

### Interaction with stress-responsive genes

Flavonoid biosynthesis is tightly regulated in response to a variety of abiotic stresses, including salt [128], drought [129], heavy metal exposure such as lead [130], magnesium deficiency [131], and microplastic contamination [132]. These environmental challenges typically upregulate flavonoid biosynthetic pathways, driving the accumulation of specific metabolites that mitigate oxidative damage, stabilize cellular structures, and enhance overall stress tolerance. Intriguingly, the interaction of multiple stresses often elicits synergistic effects, further amplifying flavonoid accumulation. For instance, combined salt and drought stress in maize markedly elevated flavonoid levels, thereby reinforcing antioxidant defenses and maintaining ionic homeostasis [133]. Similarly, cold and ultraviolet (UV) radiation in *A. thaliana* triggered a pronounced increase in flavonoid content, underscoring the adaptive significance of these compounds under complex stress scenarios [134]. Together, these findings highlight the critical role of flavonoids as multifunctional mediators of plant resilience, providing both structural protection and redox balance during abiotic stress.

Moreover, flavonoid biosynthesis is intricately linked to the expression of stress-responsive genes, often orchestrated by overlapping transcriptional networks (Table 4). When cold stress coincides with other abiotic factors, such as UV radiation or salinity, the regulatory influence on flavonoid metabolism is significantly amplified. For instance, in *A. thaliana*, the combination of cold and UV stress modulated the expression of key genes involved in flavonoid biosynthesis, reflecting a coordinated regulatory response to concurrent stress signals [134]. In pepper (*Capsicum annuum*), simultaneous cold and salt stress markedly elevated antioxidant glycoside levels, further illustrating the dynamic interplay between transcriptional regulation and metabolic adaptation [8]. These insights underscore the pivotal role of flavonoids in mediating plant resilience by integrating diverse environmental cues into finely tuned metabolic responses (Fig. 3b). A deeper understanding of these regulatory networks will provide critical opportunities to optimize flavonoid pathways and enhance crop resilience in increasingly complex stress environments.

**Table 4:** Genes involved in flavonoid-mediated stress response under cold stress

**Gene**	**Function**	**References**
*McWRKY43*	Regulates flavonoid biosynthesis to improve cold stress tolerance by enhancing antioxidant defense.	[135]
*CsUGT78A14*	Increases flavonoid glycosides, aiding ROS scavenging under cold stress.	[82]
*OsDfr & OsAns*	Activates flavonoid synthesis in response to dehydration, high salt, and ABA, contributing to stress tolerance.	[87]
*HSP70, ERD,* and *CBF*	Activates antioxidant and osmotic responses, modulating flavonoid pathways to enhance resilience under cold stress.	[136]
*F3H* and *PAL*	Key flavonoid biosynthesis genes that increase cold tolerance by stabilizing cellular redox balance.	[137]
*ANS* and *CHS*	Upregulates pathways linked to cold-responsive metabolites in mango, enhancing tolerance to low temperatures.	[138]
*FLS* & *CHI*	Flavonoid pathway genes that facilitate ROS detoxification and cellular stability in wheat.	[139]
*NAC* and *MYB*	Control the expression of flavonoid and lignin pathways under stress conditions, contributing to cold adaptation.	[97].

### Integration with calcium signaling

Calcium signaling is another pathway that interacts with flavonoids during cold stress. Flavonoids help modulate calcium levels in cells, and cold stress activates calcium channels, critical for activating downstream signaling cascades that confer cold tolerance [140]. Cold stress treatment and calcium inhibitor assays showed that Ca^2+^ inhibitor pretreatment blocked Ca^2+^ signaling and impaired the cold-response network, significantly inhibiting the expression of *CsCBFs* genes and leading to a significant accumulation of catechins [140, 141]. This suggests that higher flavonoid accumulation plays a positive role in the cold response, highlighting the multifaceted role of flavonoids in integrating various signaling pathways to enhance stress resilience (Fig. 3c).

### Applications in horticultural crops

Advancements in genetic engineering and breeding strategies offer promising avenues for enhancing cold tolerance and improving crop quality in horticultural crops. This section focuses on genetic engineering, breeding strategies to increase flavonoid content, and practical applications through case studies in horticultural crops.

### Enhancing cold tolerance through genetic engineering

Genetic engineering is a critical tool for enhancing cold tolerance in horticultural crops, allowing for targeted manipulation of stress-responsive genes to improve resilience against chilling temperatures. Transgenic approaches have already produced cold-tolerant crops by modulating key genes associated with cold stress responses, providing a sustainable solution to mitigate the adverse effects of cold on productivity [142]. Among these technologies, CRISPR/Cas9 has emerged as particularly transformative, facilitating precise editing of genes involved in flavonoid biosynthesis, a pathway closely linked to cold stress resilience.

Studies have demonstrated that increasing specific flavonoid compounds, such as anthocyanins and quercetin, via CRISPR/Cas9 and transgenic methods enhances ROS scavenging capacity and strengthens antioxidant defenses, bolstering stress tolerance. Integrating high-throughput phenotyping and genomic selection into these breeding programs further streamlines trait selection for cold tolerance and flavonoid enhancement [143, 144]. Emerging tools, including machine learning and metabolomics, optimize these traits with greater speed and precision, advancing the breeding of cold-tolerant horticultural varieties [145, 146].

Recent advancements in CRISPR/Cas9 have expanded the potential for directly enhancing flavonoid pathways. By targeting specific genes such as chalcone synthase (*CHS*) and flavanone 3-hydroxylase (*F3H*), researchers have achieved increased flavonoid accumulation and improved antioxidant profiles in species like *Torenia fournieri* and *Nicotiana tabacum* [147, 148]. Similarly, CRISPR/Cas9-mediated mutagenesis of *FtMYB45* in Tartary buckwheat significantly promotes flavonoid biosynthesis, enhancing stress resilience [149]. Applications extend to major crops as well: multiplex CRISPR/Cas9 editing in soybean has elevated isoflavone content and viral resistance, while targeted editing of *MdPGT1* in apple has reduced phloridzin levels without affecting growth, underscoring CRISPR/Cas9’s capacity for precise metabolic engineering [150, 151]. These advances underscore the versatility of CRISPR/Cas9 in modifying flavonoid biosynthesis pathways, reinforcing cold tolerance and expanding the potential for horticultural crops to adapt to environmental challenges. Although CRISPR/Cas9 has transformed genome editing, off-target effects remain a critical concern, as single-guide RNAs (sgRNAs) may inadvertently bind to nontarget sites, leading to unintended mutations [152]. To mitigate these risks, advancements such as high-fidelity Cas9 variants (e.g. HiFi Cas9, eSpCas9), optimized sgRNA design tools, and tissue-specific delivery systems have been developed [153–155]. Methods like GUIDE-seq and Digenome-seq enable precise identification of off-target events, ensuring genomic integrity [155]. These strategies provide a robust framework for safely applying CRISPR/Cas9 to enhance flavonoid biosynthesis, supporting its role in improving plant resilience and stress tolerance.

### Breeding strategies to increase flavonoid content

Breeding strategies aimed at increasing flavonoid content in horticultural crops hold significant promise for improving plant stress responses and overall crop quality [156]. By selecting cultivars with naturally higher flavonoid content, breeders can develop varieties with enhanced stress tolerance and nutritional value [157]. Understanding the biosynthetic pathways of flavonoids and their interactions with regulatory networks enables breeders to target specific genes for enhancing flavonoid accumulation in crops [158]. Recent advancements in flavonoid biosynthesis and cold tolerance breeding have been significantly bolstered by CRISPR/Cas9 genome editing, which enables precise and targeted trait enhancement in horticultural crops. Through CRISPR/Cas9, researchers have successfully modified key genes to increase flavonoid levels, improving cold tolerance and antioxidant capacity. This approach accelerates breeding outcomes by enabling the simultaneous editing of multiple genes, facilitating more robust flavonoid profiles and resilience to abiotic stresses [159–161]. These breeding strategies pave the way for the development of horticultural crops with improved nutritional profiles and enhanced resilience to environmental stresses.

### Practical applications and case studies

Practical applications of genetic engineering and breeding strategies in horticultural crops have yielded tangible results in enhancing stress tolerance and crop quality [162]. Case studies showcasing the successful implementation of genetic engineering techniques, such as the introgression of stress-responsive genes, have led to the development of crop varieties with enhanced cold tolerance [163]. Additionally, the application of biotechnology tools, such as genome editing and transgenic technology, has enabled the improvement of crop varieties for enhanced cold tolerance without compromising yield [164]. These practical applications underscore the potential of genetic engineering in revolutionizing horticultural crop production and sustainability.

**Figure 4:**
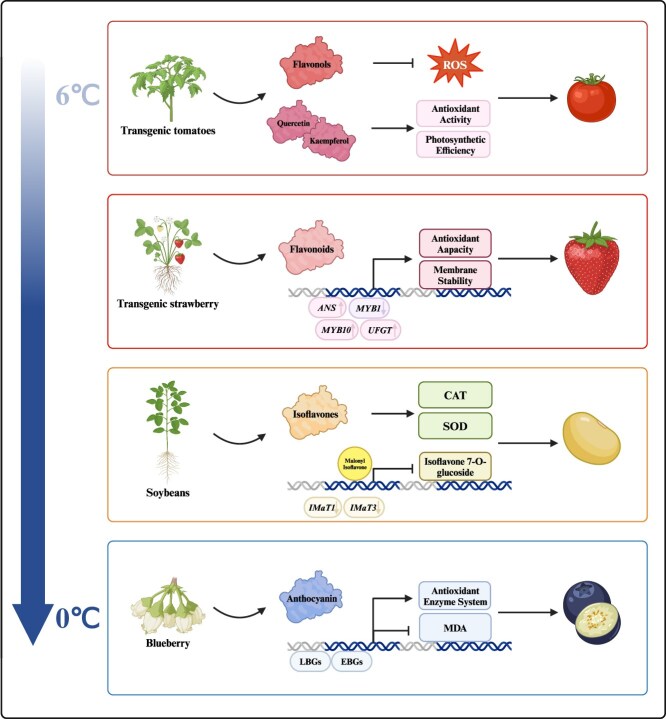
Functions of flavonoids in horticultural plants under cold stress. This figure illustrates the roles of various flavonoids in enhancing the cold resistance of horticultural plants. In tomatoes, flavonols such as quercetin and kaempferol are synthesized in response to cold stress, playing a crucial role in scavenging ROS, thus enhancing antioxidant activity and photosynthetic efficiency. In strawberries, the synthesis of flavonoids under cold stress is regulated by genes such as anthocyanidin synthase (*ANS*), MYB transcription factors (*MYB1*, *MYB10*), and UDP-glucose: flavonoid 3-O-glucosyltransferase (*UFGT*). These flavonoids enhance the plant’s antioxidant capacity and membrane stability, ensuring better survival under cold conditions. In soybeans, cold stress activates isoflavone biosynthetic pathways involving genes such as *IMaT1* and *IMaT3*. Isoflavones improve the activity of antioxidant enzymes like CAT and SOD and increase the accumulation of isoflavone 7-O-glucoside, contributing to enhanced stress tolerance. In blueberries, cold stress induces the expression of early (EBGs) and late (LBGs) flavonoid biosynthetic genes, leading to the accumulation of anthocyanins. These compounds enhance the antioxidant enzyme system and reduce MDA levels, thereby protecting the plant from oxidative damage. *IMaT1*, Invasive meningioma associated transcript 1; *IMaT3*, Invasive meningioma associated transcript 3. Figure created with Biorender (https://www.biorender.com/).

Building on these genetic engineering and biotechnological approaches, recent advancements in flavonoid biosynthesis research have demonstrated the critical role of specific flavonoids in enhancing cold tolerance across a range of horticultural crops. For instance, in transgenic tomatoes, increased biosynthesis of flavonols, such as quercetin and kaempferol, enhances antioxidant activity and supports photosynthetic efficiency under cold stress by scavenging ROS, resulting in improved cold tolerance [82, 165–167]. Similarly, in strawberries, the upregulation of anthocyanin-related genes (e.g. *MYB10*, *ANS*, and *UFGT*) under cold stress conditions leads to higher flavonoid accumulation, which strengthens antioxidant capacity and stabilizes cellular membranes [168–170]. In soybeans, isoflavones activate antioxidant enzymes, such as catalase and superoxide dismutase (SOD), to manage ROS, with key enzymes like *GmIMaT1* and *GmIMaT3* modulating isoflavone glucoside levels to provide a protective response to cold [171–173]. Blueberries, naturally high in anthocyanins, show strong resilience to cold as anthocyanins boost antioxidant enzyme activity, reduce oxidative damage, and maintain cellular integrity; exogenous anthocyanin applications have been shown to further enhance these effects [174–177]. These case studies underscore the potential of flavonoid-targeted genetic and biotechnological interventions to improve cold tolerance in horticultural crops, providing a foundation for breeding programs focused on stress resilience (Fig. 4).

These studies collectively highlight the significant role of flavonoids in conferring cold tolerance to horticultural plants. By enhancing antioxidant capacity, stabilizing cellular membranes, and modulating stress-responsive gene expression, flavonoids contribute to the resilience of crops to cold stress. The integration of genetic engineering and breeding strategies in horticultural crop improvement offers innovative solutions for enhancing cold tolerance, increasing flavonoid content, and improving overall crop quality. By harnessing the power of biotechnology and targeted breeding approaches, horticultural crops can be fortified against environmental stresses, ensuring food security and sustainable agriculture in the face of changing climatic conditions.

## Conclusions and perspective

The field of flavonoid research holds immense potential for further exploration and innovation. This section reviews the knowledge gaps in flavonoid research, proposes potential strategies for future investigations, and discusses the implications of flavonoid research for sustainable horticulture.

## Knowledge gaps in flavonoid research

Despite significant advancements, several knowledge gaps persist, hindering a comprehensive understanding of the full spectrum of flavonoid functions and applications. Key gaps include inconsistent nomenclature, inappropriate analytical methods, variability in existing flavonoid databases, and inadequate consideration of test material design in intervention trials [178]. A primary challenge lies in the inconsistent nomenclature used to classify flavonoids, leading to ambiguities across studies. Compounding this is the use of varied and sometimes inappropriate analytical methods that fail to capture the specific contributions of individual flavonoid compounds. Further complicating research efforts, existing databases lack standardized data on flavonoid forms and do not consistently account for food preparation or geographic variability, which affects flavonoid content and bioavailability [178]. Additionally, understanding the *in vivo* biotransformation mechanisms of dietary flavonoids remains limited, constraining our knowledge of their activities and practical applications [179]. Addressing these gaps is crucial for advancing flavonoid research and unlocking the full potential of these bioactive compounds.

## Potential strategies for future research

Future research in flavonoid studies should focus on bridging existing knowledge gaps and exploring novel avenues. Strategies may include conducting further field studies to elucidate the metabolic pathways and health benefits of flavonoids [180]. Optimizing delivery strategies, exploring synergistic combinations, and conducting comprehensive field intervention trials are essential for advancing our understanding of flavonoids and their therapeutic potential [181]. Leveraging advanced technologies, such as genome editing and nanoparticle-based delivery systems, can offer innovative approaches to enhance the bioavailability and efficacy of flavonoids [182]. Collaborative efforts among multidisciplinary research teams and the integration of computational modeling can provide valuable insights into flavonoid activities and mechanisms [183]. By adopting these advanced strategies, researchers can unlock new potentials of flavonoids in enhancing stress tolerance in plants and improving human health. The integration of field trials and cutting-edge technologies will help translate laboratory findings into practical applications, fostering the development of robust horticultural crops and novel therapeutic agents.

## Implications for sustainable horticulture

The implications of flavonoid research extend beyond scientific discovery to practical applications in sustainable horticulture. By leveraging the antioxidant and stress-mitigating properties of flavonoids, researchers can develop targeted strategies to enhance crop resilience against abiotic stresses such as drought, salinity, and extreme temperatures, alongside cold stress. Increasing flavonoid levels in crops can also lead to improved nutritional profiles, offering health benefits to consumers and adding value to horticultural products. In breeding and genetic programs, incorporating flavonoid biosynthesis pathways could result in crop varieties with enhanced resistance to multiple stresses, reducing the need for chemical inputs such as pesticides and fertilizers, thus promoting eco-friendly and cost-effective production systems. Furthermore, integrating flavonoid-rich plant extracts and nanoparticles into crop management can offer eco-friendly solutions for pest control and disease management, promoting sustainable crop production [184]. These advancements suggest that flavonoid research has the potential to revolutionize horticultural practices. By contributing to more resilient, nutrient-rich, and environmentally friendly crop varieties, flavonoid-based innovations support food security, environmental sustainability, and human health. In the long term, these strategies could play a significant role in achieving a sustainable and climate-resilient horticultural industry. Future research should prioritize uncovering the molecular mechanisms through which specific flavonoids enhance cold tolerance, focusing on their regulation of stress-responsive genes and signaling pathways. Functional validation using CRISPR/Cas9-mediated editing or overexpression of key biosynthetic genes, such as those involved in flavonol and anthocyanin synthesis, will be critical to establishing their roles. Furthermore, investigating their interplay with phytohormones and other metabolic pathways could reveal synergistic mechanisms that bolster plant resilience. Coupling these insights with field-based validation will advance the development of stress-resilient horticultural crops tailored to diverse environmental challenges.

In conclusion, this review underscores the central role of flavonoids in enhancing cold tolerance in horticultural crops. By exploring their antioxidant properties, membrane-stabilizing effects, and regulation of cold-responsive genes, flavonoids are revealed as vital contributors to plant resilience under low temperatures. However, to fully leverage these benefits, several key knowledge gaps must be addressed, such as the need for standardized analytical methods and consistent nomenclature across studies, as well as deeper insights into the molecular pathways influenced by flavonoids during cold stress. Future research should focus on integrating advanced biotechnological tools like CRISPR/Cas9 and multiomics approaches to manipulate flavonoid pathways, thereby improving cold tolerance in horticultural crops. Such innovations not only have the potential to strengthen crop resilience in changing climates but also contribute to sustainable agricultural practices by reducing the need for chemical inputs. Through these interdisciplinary and application-focused strategies, flavonoid research can pave the way toward robust, cold-tolerant horticultural systems, supporting both food security and environmental sustainability.

## Data Availability

The authors confirm that all data in this study are available and can be found in this article.

## References

[1] Li R, Zhang L, Wang L. et al. Reduction of tomato-plant chilling tolerance by CRISPR-Cas9-mediated SlCBF1 mutagenesis. J Agric Food Chem. 2018;66:9042–51 30096237 10.1021/acs.jafc.8b02177

[2] Hao W, Arora R, Yadav AK. et al. Freezing tolerance and cold acclimation in guava (*Psidium guajava* L.). HortScience. 2009;44:1258–66

[3] Yadav SK . Cold stress tolerance mechanisms in plants. A review. Agron Sustain Dev. 2010;30:515–27

[4] Yuwansiri R, Park E-J, Jeknić Z. et al. Enhancing cold tolerance in plants by genetic engineering of glycinebetaine synthesis. In: Li PH, Tapio Palva E (eds), Plant Cold Hardiness: Gene Regulation and Genetic Engineering. Boston, MA: Springer, 2002, 259–75

[5] Juurakko CL, diCenzo GC, Walker VK. Cold acclimation and prospects for cold-resilient crops. Plant Stress. 2021;2:100028

[6] Jahed KR, Saini AK, Sherif SM. Coping with the cold: unveiling cryoprotectants, molecular signaling pathways, and strategies for cold stress resilience. Front Plant Sci. 2023;14:1246093 37649996 10.3389/fpls.2023.1246093 PMC10465183

[7] Theocharis A, Bordiec S, Fernandez O. et al. *Burkholderia phytofirmans* PsJN primes *Vitis vinifera* L. and confers a better tolerance to low nonfreezing temperatures. Mol Plant-Microbe Interact. 2012;25:241–9 21942451 10.1094/MPMI-05-11-0124

[8] Genzel F, Dicke MD, Junker-Frohn LV. et al. Impact of moderate cold and salt stress on the accumulation of antioxidant flavonoids in the leaves of two *Capsicum* cultivars. J Agric Food Chem. 2021;69:6431–43 34081868 10.1021/acs.jafc.1c00908

[9] Peng Z, Wang Y, Zuo W-T. et al. Integration of metabolome and transcriptome studies reveals flavonoids, abscisic acid, and nitric oxide comodulating the freezing tolerance in *Liriope spicata*. Front Plant Sci. 2022;12:764625 35154173 10.3389/fpls.2021.764625 PMC8828910

[10] Yu L, Sun Y, Zhang X. et al. ROS1 promotes low temperature-induced anthocyanin accumulation in apple by demethylating the promoter of anthocyanin-associated genes. Hortic Res. 2022;9:uhac007 35147161 10.1093/hr/uhac007 PMC9123231

[11] Wang J, Zhang Y, Wang J. et al. *SlGAD2* is the target of SlTHM27, positively regulates cold tolerance by mediating anthocyanin biosynthesis in tomato. Hortic Res. 2024;11:uhae096 38855415 10.1093/hr/uhae096 PMC11161262

[12] Cai M, Huang J, Chen M. et al. The role and synthesis mechanism of anthocyanins in *Sphagneticola trilobata* stems under low temperature. Biol Invasions. 2024;26:2851–67

[13] Wang H, Wei X, Mo C. et al. Integrated full-length transcriptome and metabolome analysis reveals the defence response of melon to gummy stem blight. Plant Cell Environ. 2024;47:1997–2010 38379450 10.1111/pce.14865

[14] Xiao P, Qu J, Wang Y. et al. Transcriptome and metabolome atlas reveals contributions of sphingosine and chlorogenic acid to cold tolerance in *Citrus*. Plant Physiol. 2024;196:kiae327 10.1093/plphys/kiae327 38875157

[15] Mei C, Yang J, Mei Q. et al. MdNAC104 positively regulates apple cold tolerance via CBF-dependent and CBF-independent pathways. Plant Biotechnol J. 2023;21:2057–73 37387580 10.1111/pbi.14112 PMC10502760

[16] Zou S-C, Zhuo M-G, Abbas F. et al. ROS- and CBF- mediated pathways are involved in chlorophyll degradation and anthocyanin accumulation enhanced by cool temperatures in ripening litchi fruits. Postharvest Biol Technol. 2024;212:112888

[17] Lin S, Singh RK, Moehninsi. et al. R2R3-MYB transcription factors, StmiR858 and sucrose mediate potato flavonol biosynthesis. Hortic Res. 2021;8:25 33518700 10.1038/s41438-021-00463-9 PMC7847999

[18] Zheng L, Li B, Zhang G. et al. Jasmonate enhances cold acclimation in jojoba by promoting flavonol synthesis. Hortic Res. 2024;11:uhae125 38966867 10.1093/hr/uhae125 PMC11220180

[19] Tian L-x, Li J. The effects of exogenous ABA applied to maize (*Zea mays* L.) roots on plant responses to chilling stress. Acta Physiol Plant. 2018;40:77

[20] Tian K, Li Q, Zhang X. et al. Analysis of the expression and function of the CBL-CIPK network and MAPK cascade genes in *Kandelia obovata* seedlings under cold stress. Front Mar Sci. 2023;10:1113278

[21] Zhang M, Li M, Fu H. et al. Transcriptomic analysis unravels the molecular response of *Lonicera japonica* leaves to chilling stress. Front Plant Sci. 2022;13:1092857 36618608 10.3389/fpls.2022.1092857 PMC9815118

[22] An Z, Yang Z, Zhou Y. et al. OsJRL negatively regulates rice cold tolerance via interfering phenylalanine metabolism and flavonoid biosynthesis. Plant Cell Environ. 2024;47:4071–85 38884189 10.1111/pce.15005

[23] Liu Y, Tikunov Y, Schouten RE. et al. Anthocyanin biosynthesis and degradation mechanisms in *Solanaceous* vegetables: a review. Front Chem. 2018;6:52 29594099 10.3389/fchem.2018.00052 PMC5855062

[24] Zuk M, Działo M, Richter D. et al. Chalcone synthase (CHS) gene suppression in flax leads to changes in wall synthesis and sensing genes, cell wall chemistry and stem morphology parameters. Front Plant Sci. 2016;7:894 27446124 10.3389/fpls.2016.00894 PMC4919909

[25] Mei X, Wan S, Lin C. et al. Integration of metabolome and transcriptome reveals the relationship of benzenoid-phenylpropanoid pigment and aroma in purple tea flowers. Front Plant Sci. 2021;12:762330 34887890 10.3389/fpls.2021.762330 PMC8649654

[26] Wang L, Sun X, Weiszmann J. et al. System-level and granger network analysis of integrated proteomic and metabolomic dynamics identifies key points of grape berry development at the interface of primary and secondary metabolism. Front Plant Sci. 2017;8:1066 28713396 10.3389/fpls.2017.01066 PMC5491621

[27] Gupta OP, Nigam D, Dahuja A. et al. Regulation of isoflavone biosynthesis by miRNAs in two contrasting soybean genotypes at different seed developmental stages. Front Plant Sci. 2017;8:567 28450878 10.3389/fpls.2017.00567 PMC5390031

[28] Zhao Q, Zhang Y, Wang G. et al. A specialized flavone biosynthetic pathway has evolved in the medicinal plant, *Scutellaria baicalensis*. Sci Adv. 2016;2:e1501780 27152350 10.1126/sciadv.1501780 PMC4846459

[29] Liu W, Feng Y, Yu S. et al. The flavonoid biosynthesis network in plants. Int J Mol Sci. 2021;23:12824 10.3390/ijms222312824 PMC8657439 34884627

[30] Fraser CM, Chapple C. The phenylpropanoid pathway in Arabidopsis. Arabidopsis Book. 2011;9:e0152-e 22303276 10.1199/tab.0152 PMC3268504

[31] Miyahara T, Sakiyama R, Ozeki Y. et al. Acyl-glucose-dependent glucosyltransferase catalyzes the final step of anthocyanin formation in Arabidopsis. J Plant Physiol. 2013;170:619–24 23298714 10.1016/j.jplph.2012.12.001

[32] D’Auria JC, Reichelt M, Luck K. et al. Identification and characterization of the BAHD acyltransferase malonyl CoA: anthocyanidin 5-O-glucoside-6″-O-malonyltransferase (At5MAT) in *Arabidopsis thaliana*. FEBS Lett. 2007;581:872–8 17292360 10.1016/j.febslet.2007.01.060

[33] Luo J, Nishiyama Y, Fuell C. et al. Convergent evolution in the BAHD family of acyl transferases: identification and characterization of anthocyanin acyl transferases from *Arabidopsis thaliana*. Plant J Cell Mol Biol. 2007;50:678–95 10.1111/j.1365-313X.2007.03079.x 17425720

[34] Zhang P, Lu S, Liu Z. et al. Transcriptomic and metabolomic profiling reveals the effect of LED light quality on fruit ripening and anthocyanin accumulation in Cabernet Sauvignon grape. Front Nutr. 2021;8:790697 34970581 10.3389/fnut.2021.790697 PMC8713590

[35] Su M, Wang S, Liu W. et al. MdJa2 participates in the Brassinosteroid signaling pathway to regulate the synthesis of anthocyanin and proanthocyanidin in red-fleshed apple. Front Plant Sci. 2022;13:830349 35615132 10.3389/fpls.2022.830349 PMC9125324

[36] Yang L, Yang Q, Zhang L. et al. Integrated metabolomics and transcriptomics analysis of flavonoid biosynthesis pathway in *Polygonatum cyrtonema* Hua. Molecules. 2024;29:2248 38792110 10.3390/molecules29102248 PMC11124200

[37] Zhang D, Wang S, Lin L. et al. Integrative analysis of metabolome and transcriptome reveals the mechanism of flavonoid biosynthesis in *Lithocarpus polystachyus* Rehd. ACS Omega. 2022;7:19437–53 35722012 10.1021/acsomega.2c01125 PMC9202069

[38] Meng L, Zhang S, Bai X. et al. Transcriptomic and non-targeted metabolomic analyses reveal the flavonoid biosynthesis pathway in *Auricularia cornea*. Molecules. 2022;27:2334 35408732 10.3390/molecules27072334 PMC9000485

[39] Li Y, Zhang J, Wang S. et al. Integrative transcriptomic and metabolomic analyses reveal the flavonoid biosynthesis of *Pyrus hopeiensis* flowers under cold stress. Hortic Plant J. 2023;9:395–413

[40] Liu Y, Tang N, Lin D. et al. Integration of multi-omics analyses highlights the secondary metabolism response of tomato fruit to low temperature storage. Food Res Int. 2023;173:113316 37803628 10.1016/j.foodres.2023.113316

[41] Zhao Y, Zhang G, Tang Q. et al. *EbMYBP1*, a R2R3-MYB transcription factor, promotes flavonoid biosynthesis in *Erigeron breviscapus*. Front Plant Sci. 2022;13:946827 35968130 10.3389/fpls.2022.946827 PMC9366350

[42] Yan H, Zheng W, Wang Y. et al. Integrative metabolome and transcriptome analysis reveals the regulatory network of flavonoid biosynthesis in response to MeJA in *Camelliavietnamensis* Huang. Int J Mol Sci. 2022;23:9370 36012624 10.3390/ijms23169370 PMC9409299

[43] An JP, Wang XF, Zhang XW. et al. An apple MYB transcription factor regulates cold tolerance and anthocyanin accumulation and undergoes MIEL1-mediated degradation. Plant Biotechnol J. 2020;18:337–53 31250952 10.1111/pbi.13201 PMC6953192

[44] Song T, Li K, Wu T. et al. Identification of new regulators through transcriptome analysis that regulate anthocyanin biosynthesis in apple leaves at low temperatures. PLoS One. 2019;14:e0210672 30695036 10.1371/journal.pone.0210672 PMC6350969

[45] An JP, Li R, Qu FJ. et al. R2R3-MYB transcription factor MdMYB23 is involved in the cold tolerance and proanthocyanidin accumulation in apple. Plant J. 2018;96:562–77 30054966 10.1111/tpj.14050

[46] Nan W, Changzhi Q, Shenghui J. et al. The proanthocyanidin-specific transcription factor MdMYBPA1 initiates anthocyanin synthesis under low temperature conditions in red-fleshed apple. Plant J Cell Mol Biol. 2018;96:39–55 10.1111/tpj.14013 29978604

[47] Wang Y, Li S, Shi Y. et al. The R2R3 MYB *Ruby1* is activated by two cold responsive ethylene response factors, via the retrotransposon in its promoter, to positively regulate anthocyanin biosynthesis in citrus. Plant J. 2024;119:1433–48 38922743 10.1111/tpj.16866 PMC13087489

[48] Mao W, Han Y, Chen Y. et al. Low temperature inhibits anthocyanin accumulation in strawberry fruit by activating FvMAPK3-induced phosphorylation of FvMYB10 and degradation of chalcone synthase 1. Plant Cell. 2022;34:1226–49 35018459 10.1093/plcell/koac006 PMC8972286

[49] Xing D, Jin D, Zheng T. et al. CsMIEL1 effectively inhibits the accumulation of anthocyanins under low temperatures in tea plants (*Camellia sinensis*). Plant Physiol Biochem. 2024;211:108726 38744083 10.1016/j.plaphy.2024.108726

[50] Xu A, Cheng F, Zhou S. et al. Chilling-induced H_2_O_2_ signaling activates the antioxidant enzymes in alleviating the photooxidative damage caused by loss of function of 2-Cys peroxiredoxin in watermelon. Plant Stress. 2022;6:100108

[51] Jie Z, Kai C, Xinyue L. et al. Exogenous abscisic acid and sodium nitroprusside regulate flavonoid biosynthesis and photosynthesis of *Nitraria tangutorum* Bobr in alkali stress. Front Plant Sci. 2023;14:1118984 37008502 10.3389/fpls.2023.1118984 PMC10057120

[52] Song Z, Lai X, Chen H. et al. MaC2H2-like regulates chilling stress response of ‘Fenjiao’ banana by modulating flavonoid synthesis and fatty acid desaturation. Food Chem. 2023;419:136089 37023674 10.1016/j.foodchem.2023.136089

[53] Luo H, Guan Y, Zhang Z. et al. *FveDREB1B* improves cold tolerance of woodland strawberry by positively regulating *FveSCL23* and *FveCHS*. Plant Cell Environ. 2024;47:4630–50 39051467 10.1111/pce.15052

[54] Sivankalyani V, Feygenberg O, Diskin S. et al. Increased anthocyanin and flavonoids in mango fruit peel are associated with cold and pathogen resistance. Postharvest Biol Technol. 2016;111:132–9

[55] Sun S, Qi X, Zhang Z. et al. A structural variation in the promoter of the leucoanthocyanidin reductase gene *AaLAR1* enhances freezing tolerance by modulating proanthocyanidin accumulation in kiwifruit (*Actinidia arguta*). Plant Cell Environ. 2024;47:4048–66 38884345 10.1111/pce.15003

[56] Carmona L, Alquézar B, Marques VV. et al. Anthocyanin biosynthesis and accumulation in blood oranges during postharvest storage at different low temperatures. Food Chem. 2017;237:7–14 28764055 10.1016/j.foodchem.2017.05.076

[57] Gaiotti F, Pastore C, Filippetti I. et al. Low night temperature at veraison enhances the accumulation of anthocyanins in Corvina grapes (*Vitis Vinifera* L.). Sci Rep. 2018;8:8719 29880890 10.1038/s41598-018-26921-4 PMC5992194

[58] Peng X, Wu H, Chen H. et al. Transcriptome profiling reveals candidate flavonol-related genes of *Tetrastigma hemsleyanum* under cold stress. BMC Genomics. 2019;20:687 31472675 10.1186/s12864-019-6045-y PMC6717372

[59] Li F, Guo S, Zhao Y. et al. Overexpression of a homopeptide repeat-containing bHLH protein gene (OrbHLH001) from Dongxiang wild rice confers freezing and salt tolerance in transgenic Arabidopsis. Plant Cell Rep. 2010;29:977–86 20559833 10.1007/s00299-010-0883-z

[60] Gao Y, Dong X, Wang R. et al. Exogenous calcium alleviates oxidative stress caused by salt stress in peanut seedling roots by regulating the antioxidant enzyme system and flavonoid biosynthesis. Antioxidants. 2024;13:233 38397831 10.3390/antiox13020233 PMC10886236

[61] Zhang C, Dai Z, Ferrier T. et al. MYB24 orchestrates terpene and flavonol metabolism as light responses to anthocyanin depletion in variegated grape berries. PlantCell. 2023;35:4238–65 10.1093/plcell/koad228 PMC10689149 37648264

[62] Zhang X, Li L, He Y. et al. The CsHSFA-CsJAZ6 module-mediated high temperature regulates flavonoid metabolism in *Camellia sinensis*. Plant Cell Environ. 2023;46:2401–18 37190917 10.1111/pce.14610

[63] Shen Y, Sun T, Pan Q. et al. RrMYB5- and RrMYB10-regulated flavonoid biosynthesis plays a pivotal role in feedback loop responding to wounding and oxidation in *Rosa rugosa*. Plant Biotechnol J. 2019;17:2078–95 30951245 10.1111/pbi.13123 PMC6790370

[64] Zhang X, Ahmad N, Zhang Q. et al. Safflower flavonoid 3′5′hydroxylase promotes methyl Jasmonate-induced anthocyanin accumulation in transgenic plants. Molecules. 2023;28:3205 37049967 10.3390/molecules28073205 PMC10095914

[65] Xu H, Wang N, Wang Y. et al. Overexpression of the transcription factor MdbHLH33 increases cold tolerance of transgenic apple callus. Plant Cell Tissue Organ Cult (PCTOC). 2018;134:131–40

[66] Lin CY, Chen PY, Hsu HJ. et al. The citrus flavonoid nobiletin downregulates angiopoietin-like protein 3 (ANGPTL3) expression and exhibits lipid-modulating effects in hepatic cells and adult zebrafish models. Int J Mol Sci. 2022;23:12485 36293338 10.3390/ijms232012485 PMC9604320

[67] Feng S, Yao Y-T, Wang B-B. et al. Flavonoids are involved in salt tolerance through ROS scavenging in the halophyte *Atriplex canescens*. Plant Cell Rep. 2024;43:5 10.1007/s00299-023-03087-6 38127154

[68] Jeevan SP, Rajendra S, Banerjee R. et al. Seed birth to death: dual functions of reactive oxygen species in seed physiology. Ann Bot. 2015;116:663–8 26271119 10.1093/aob/mcv098 PMC4578000

[69] Ayala A, Muñoz MF, Argüelles S. Lipid peroxidation: production, metabolism, and signaling mechanisms of malondialdehyde and 4-hydroxy-2-nonenal. Oxidative Med Cell Longev. 2014;2014:360438 10.1155/2014/360438 PMC4066722 24999379

[70] Cho CH, Jang H, Lee M. et al. Sea buckthorn (*Hippophae rhamnoides* L.) leaf extracts protect neuronal PC-12 cells from oxidative stress. J Microbiol Biotechnol. 2017;27:1257–65 28535611 10.4014/jmb.1704.04033

[71] Zhan X, Shen Q, Chen J. et al. Rice sulfoquinovosyltransferase SQD2.1 mediates flavonoid glycosylation and enhances tolerance to osmotic stress. Plant Cell Environ. 2019;42:2215–30 30942482 10.1111/pce.13554

[72] Zhang L, Wang L, Fang Y. et al. Phosphorylated transcription factor PuHB40 mediates ROS-dependent anthocyanin biosynthesis in pear exposed to high light. Plant Cell. 2024;36:koae167 10.1093/plcell/koae167 PMC11371158 38842382

[73] Jeong J-M, Choi C-H, Kang S-K. et al. Antioxidant and chemosensitizing effects of flavonoids with hydroxy and/or methoxy groups and structure-activity relationship. J Pharm Pharm Sci. 2007;10:537–46 18261373 10.18433/j3kw2z

[74] Guo W, Beta T. Phenolic acid composition and antioxidant potential of insoluble and soluble dietary fibre extracts derived from select whole-grain cereals. Food Res Int. 2013;51:518–25

[75] Mi W, Liu Z, Jin J. et al. Comparative proteomics analysis reveals the molecular mechanism of enhanced cold tolerance through ROS scavenging in winter rapeseed (*Brassica napus* L.). PLoS One. 2021;16:e0243292 33434207 10.1371/journal.pone.0243292 PMC7802968

[76] Liu L, Si L, Zhang L. et al. Metabolomics and transcriptomics analysis revealed the response mechanism of alfalfa to combined cold and saline-alkali stress. Plant J Cell Mol Biol. 2024;119:1900–19 10.1111/tpj.16896 38943631

[77] Sun S, Fang J, Lin M. et al. Comparative metabolomic and transcriptomic studies reveal key metabolism pathways contributing to freezing tolerance under cold stress in kiwifruit. Front Plant Sci. 2021;12:628969 34140959 10.3389/fpls.2021.628969 PMC8204810

[78] Song Y, Feng J, Liu D. et al. Different phenylalanine pathway responses to cold stress based on metabolomics and transcriptomics in Tartary buckwheat landraces. J Agric Food Chem. 2022;70:687–98 34989558 10.1021/acs.jafc.1c06915

[79] Li Y, Tian Q, Wang Z. et al. Integrated analysis of transcriptomics and metabolomics of peach under cold stress. Front Plant Sci. 2023;14:1153902 37051086 10.3389/fpls.2023.1153902 PMC10083366

[80] Zhu Z-P, Yu J-X, Liu F-F. et al. *AeWRKY32* from okra regulates anthocyanin accumulation and cold tolerance in *Arabidopsis*. J Plant Physiol. 2023;287:154062 37540924 10.1016/j.jplph.2023.154062

[81] Qiu Z, Wang X, Gao J. et al. The tomato *Hoffman's Anthocyaninless* gene encodes a bHLH transcription factor involved in anthocyanin biosynthesis that is developmentally regulated and induced by low temperatures. PLoS One. 2016;11:e0151067 26943362 10.1371/journal.pone.0151067 PMC4778906

[82] Zhao M, Jin J, Gao T. et al. Glucosyltransferase CsUGT78A14 regulates flavonols accumulation and reactive oxygen species scavenging in response to cold stress in *Camellia sinensis*. Front Plant Sci. 2019;10:1675 31929783 10.3389/fpls.2019.01675 PMC6941654

[83] Chen J, Mei S, Zheng P. et al. A multi-omics view of the preservation effect on *Camellia sinensis* leaves during low temperature postharvest transportation. LWT. 2023;178:114614

[84] Schulz E, Tohge T, Zuther E. et al. Natural variation in flavonol and anthocyanin metabolism during cold acclimation in *Arabidopsis thaliana* accessions. Plant Cell Environ. 2015;38:1658–72 25689473 10.1111/pce.12518

[85] Habibur Rahman Pramanik M, Imai R. Functional identification of a trehalose 6-phosphate phosphatase gene that is involved in transient induction of trehalose biosynthesis during chilling stress in rice. Plant Mol Biol. 2005;58:751–62 16240171 10.1007/s11103-005-7404-4

[86] Thomashow MF . PLANT COLD ACCLIMATION: freezing tolerance genes and regulatory mechanisms. Annu Rev Plant Physiol Plant Mol Biol. 1999;50:571–99 15012220 10.1146/annurev.arplant.50.1.571

[87] Ithal N, Reddy AR. Rice flavonoid pathway genes, *OsDfr* and *OsAns*, are induced by dehydration, high salt and ABA, and contain stress responsive promoter elements that interact with the transcription activator, OsC1-MYB. Plant Sci. 2004;166:1505–13

[88] Zijian X, Jiachun W, Yongbo M. et al. The bZIP transcription factor SlAREB1 regulates anthocyanin biosynthesis in response to low temperature in tomato. Plant J Cell Mol Biol. 2023;115:205–19 10.1111/tpj.16224 36999610

[89] Bhatia C, Pandey A, Gaddam SR. et al. Low temperature-enhanced flavonol synthesis requires light-associated regulatory components in *Arabidopsis thaliana*. Plant Cell Physiol. 2018;59:2099–112 30010959 10.1093/pcp/pcy132

[90] Xie XB, Li S, Zhang RF. et al. The bHLH transcription factor MdbHLH3 promotes anthocyanin accumulation and fruit colouration in response to low temperature in apples. Plant Cell Environ. 2012;35:1884–97 22519753 10.1111/j.1365-3040.2012.02523.x

[91] Fang H, Dong Y, Yue X. et al. The B-box zinc finger protein MdBBX20 integrates anthocyanin accumulation in response to ultraviolet radiation and low temperature. Plant Cell Environ. 2019;42:2090–104 30919454 10.1111/pce.13552

[92] Wu D, Wu Y, Gao R. et al. Integrated metabolomics and transcriptomics reveal the key role of flavonoids in the cold tolerance of chrysanthemum. Int J Mol Sci. 2024;25:7589 39062834 10.3390/ijms25147589 PMC11276724

[93] Zhu J, Lou H, Yan C. et al. Exogenous melatonin enhances cold tolerance by regulating the expression of photosynthetic performance, antioxidant system, and related genes in cotton. Plan Theory. 2024;13:2010 10.3390/plants13152010 PMC11314530 39124128

[94] Zhao P, Yan X, Qian C. et al. Flavonoid synthesis pathway response to low-temperature stress in a desert medicinal plant, *Agriophyllum Squarrosum* (Sandrice). Genes. 2024;15:1228 39336819 10.3390/genes15091228 PMC11431328

[95] Yang X, Han Y, Huo G. et al. Integrated transcriptomic and metabolomic analysis provides insights into cold tolerance in lettuce (*Lactuca sativa* L.). BMC Plant Biol. 2024;24:442 38778262 10.1186/s12870-024-05099-0 PMC11112944

[96] Yang X, Liu C, Li M. et al. Integrated transcriptomics and metabolomics analysis reveals key regulatory network that response to cold stress in common bean (*Phaseolus vulgaris* L.). BMC Plant Biol. 2023;23:85 36759761 10.1186/s12870-023-04094-1 PMC9909927

[97] Shomali A, Das S, Arif N. et al. Diverse physiological roles of flavonoids in plant environmental stress responses and tolerance. Plants (Basel). 2022;11:3158 36432887 10.3390/plants11223158 PMC9699315

[98] Borella M, Baghdadi A, Bertoldo G. et al. Transcriptomic and physiological approaches to decipher cold stress mitigation exerted by brown-seaweed extract application in tomato. Front Plant Sci. 2023;14:1232421 37767293 10.3389/fpls.2023.1232421 PMC10520554

[99] Zhu Y, Wang K, Wu C. et al. Effect of ethylene on cell wall and lipid metabolism during alleviation of postharvest chilling injury in peach. Cells. 2019;8:1612 31835827 10.3390/cells8121612 PMC6952997

[100] Peng T, Jia MM, Liu JH. RNAi-based functional elucidation of *PtrPRP*, a gene encoding a hybrid proline rich protein, in cold tolerance of *Poncirus trifoliata*. Front Plant Sci. 2015;6:808 26483822 10.3389/fpls.2015.00808 PMC4587090

[101] Lado J, Rodrigo MJ, López-Climent M. et al. Implication of the antioxidant system in chilling injury tolerance in the red peel of grapefruit. Postharvest Biol Technol. 2016;111:214–23

[102] Gaschler MM, Stockwell BR. Lipid peroxidation in cell death. Biochem Biophys Res Commun. 2017;482:419–25 28212725 10.1016/j.bbrc.2016.10.086 PMC5319403

[103] Poklar Ulrih N, Ota A, Šentjurc M. et al. Flavonoids and cell membrane fluidity. Food Chem. 2010;121:78–84

[104] Pang Q, Yu W, Sadeghnezhad E. et al. Omic analysis of anthocyanin synthesis in wine grape leaves under low-temperature. Sci Hortic. 2023;307:111483

[105] Taher B, Reza N, Farhang R. et al. Hydrogen sulfide and phenylalanine alleviate chilling injury in eggplant fruits during cold storage by enhancing antioxidant activities and membrane stability. J Food Process Preserv. 2021;45:e15933

[106] Chaudhuri S, Banerjee A, Basu K. et al. Interaction of flavonoids with red blood cell membrane lipids and proteins: antioxidant and antihemolytic effects. Int J Biol Macromol. 2007;41:42–8 17239435 10.1016/j.ijbiomac.2006.12.003

[107] Yan GL, Duan LL, Liu PT. et al. Transcriptional comparison investigating the influence of the addition of unsaturated fatty acids on aroma compounds during alcoholic fermentation. Front Microbiol. 2019;10:1115 31178837 10.3389/fmicb.2019.01115 PMC6538801

[108] Zhou B, Zheng B, Wu W. The ncRNAs involved in the regulation of abiotic stress-induced anthocyanin biosynthesis in plants. Antioxidants. 2023;13:55 38247480 10.3390/antiox13010055 PMC10812613

[109] Zhao Y, Zhou M, Xu K. et al. Integrated transcriptomics and metabolomics analyses provide insights into cold stress response in wheat. Crop J. 2019;7:857–66

[110] Castro-Cegrí A, Sierra S, Hidalgo-Santiago L. et al. Postharvest treatment with abscisic acid alleviates chilling injury in zucchini fruit by regulating phenolic metabolism and non-enzymatic antioxidant system. Antioxidants. 2023;12:211 36671073 10.3390/antiox12010211 PMC9854589

[111] Song Z, Lai X, Chen H. et al. Role of MaABI5-like in abscisic acid-induced cold tolerance of ‘Fenjiao’ banana fruit. Hortic Res. 2022;9:uhac130 36936195 10.1093/hr/uhac130 PMC10021067

[112] Lucho-Constantino GG, Zaragoza-Martínez F, Ponce-Noyola T. et al. Antioxidant responses under jasmonic acid elicitation comprise enhanced production of flavonoids and anthocyanins in *Jatropha curcas* leaves. Acta Physiol Plant. 2017;39:165

[113] Raza A, Charagh S, Najafi-Kakavand S. et al. Role of phytohormones in regulating cold stress tolerance: physiological and molecular approaches for developing cold-smart crop plants. Plant Stress. 2023;8:100152

[114] Bhattacharya A . Plant growth hormones in plants under low-temperature stress: a review. In: Bhattacharya A (ed.), Physiological Processes in Plants Under Low Temperature Stress. Singapore: Springer, 2022, 517–627

[115] Sang Y, Liu Y, Tang Y. et al. Transcriptome sequencing reveals mechanism of improved antioxidant capacity and maintained postharvest quality of winter jujube during cold storage after salicylic acid treatment. Postharvest Biol Technol. 2022;189:111929

[116] Niu Y, Ye L, Wang Y. et al. Transcriptome analysis reveals salicylic acid treatment mitigates chilling injury in kiwifruit by enhancing phenolic synthesis and regulating phytohormone signaling pathways. Postharvest Biol Technol. 2023;205:112483

[117] Khan N, Bano A, Ali S. et al. Crosstalk amongst phytohormones from planta and PGPR under biotic and abiotic stresses. Plant Growth Regul. 2020;90:189–203

[118] Arif Y, Sami F, Siddiqui H. et al. Salicylic acid in relation to other phytohormones in plant: a study towards physiology and signal transduction under challenging environment. Environ Exp Bot. 2020;175:104040

[119] Rahmani N, Radjabian T. Integrative effects of phytohormones in the phenolic acids production in *Salvia verticillata* L. under multi-walled carbon nanotubes and methyl jasmonate elicitation. BMC Plant Biol. 2024;24:56 38238679 10.1186/s12870-023-04719-5 PMC10797988

[120] Bhatt D, Nath M, Sharma M. et al. Role of growth regulators and phytohormones in overcoming environmental stress. In: Roychoudhury A, Tripathi DK (eds), Protective Chemical Agents in the Amelioration of Plant Abiotic Stress. Hoboken, NJ, USA: Wiley-Blackwell, 2020, 254–79

[121] Lv Z-Y, Sun W-J, Jiang R. et al. Phytohormones jasmonic acid, salicylic acid, gibberellins, and abscisic acid are key mediators of plant secondary metabolites. World J Trad Chin Med. 2021;7:307–25

[122] Wang X, Li Z, Shi Y. et al. Strigolactones promote plant freezing tolerance by releasing the WRKY41-mediated inhibition of *CBF/DREB1* expression. EMBO J. 2023;42:e112999 37622245 10.15252/embj.2022112999 PMC10548171

[123] Chi C, Chen X, Zhu C. et al. Strigolactones positively regulate HY5-dependent autophagy and the degradation of ubiquitinated proteins in response to cold stress in tomato. New Phytol. 2024;245:1106–23 39155750 10.1111/nph.20058

[124] Chi C, Xu X, Wang M. et al. Strigolactones positively regulate abscisic acid-dependent heat and cold tolerance in tomato. Hortic Res. 2021;8:237 34719688 10.1038/s41438-021-00668-y PMC8558334

[125] An S, Liu Y, Sang K. et al. Brassinosteroid signaling positively regulates abscisic acid biosynthesis in response to chilling stress in tomato. J Integr Plant Biol. 2023;65:10–24 36053143 10.1111/jipb.13356

[126] An J-P, Liu Z-Y, Zhang X-W. et al. Brassinosteroid signaling regulator BIM1 integrates brassinolide and jasmonic acid signaling during cold tolerance in apple. Plant Physiol. 2023;193:1652–74 37392474 10.1093/plphys/kiad371

[127] Bai C, Zheng Y, Watkins CB. et al. Revealing the specific regulations of Brassinolide on tomato fruit chilling injury by integrated multi-omics. Front Nutr. 2021;8:769715 34926549 10.3389/fnut.2021.769715 PMC8681340

[128] Song Z, Yang Q, Dong B. et al. Melatonin enhances stress tolerance in pigeon pea by promoting flavonoid enrichment, particularly luteolin in response to salt stress. J Exp Bot. 2022;73:5992–6008 35727860 10.1093/jxb/erac276

[129] Li Z, Han Y, Li X. et al. The phosphorylation of a WD40-repeat protein negatively regulates flavonoid biosynthesis in *Camellia sinensis* under drought stress. Hortic Res. 2024;11:uhae136 38994448 10.1093/hr/uhae136 PMC11237189

[130] Dong X, Li W, Li C. et al. Integrated transcriptomics and metabolomics revealed the mechanism of catechin biosynthesis in response to lead stress in tung tree (*Vernicia fordii*). Sci Total Environ. 2024;930:172796 38692325 10.1016/j.scitotenv.2024.172796

[131] Xiong B, Li Q, Yao J. et al. Widely targeted metabolomic profiling combined with transcriptome analysis sheds light on flavonoid biosynthesis in sweet orange 'Newhall' (*C. sinensis*) under magnesium stress. Front Plant Sci. 2023;14:1182284 37251770 10.3389/fpls.2023.1182284 PMC10216496

[132] Cui J, Li X, Gan Q. et al. Flavonoids mitigate nanoplastic stress in *Ginkgo biloba*. Plant Cell Environ. 2024, 1–22 10.1111/pce.15247 39497283

[133] Li P-c, Yang X-y, Wang H-m. et al. Metabolic responses to combined water deficit and salt stress in maize primary roots. J Integr Agric. 2021;20:109–19

[134] Schulz E, Tohge T, Winkler JB. et al. Natural variation among Arabidopsis accessions in the regulation of flavonoid metabolism and stress gene expression by combined UV radiation and cold. Plant Cell Physiol. 2021;62:502–14 33544865 10.1093/pcp/pcab013 PMC8286136

[135] Yu Q, Liu C, Sun J. et al. *McWRKY43* confers cold stress tolerance in *Michelia crassipes* via regulation of flavonoid biosynthesis. Int J Mol Sci. 2024;25:9843 39337331 10.3390/ijms25189843 PMC11432407

[136] Waititu JK, Cai Q, Sun Y. et al. Transcriptome profiling of maize (Zea mays L.) leaves reveals key cold-responsive genes, transcription factors, and metabolic pathways regulating cold stress tolerance at the seedling stage. Genes. 2021;12:1638 34681032 10.3390/genes12101638 PMC8535276

[137] Wu Y, Wei W, Pang X. et al. Comparative transcriptome profiling of a desert evergreen shrub, *Ammopiptanthus mongolicus*, in response to drought and cold stresses. BMC Genomics. 2014;15:671 25108399 10.1186/1471-2164-15-671 PMC4143566

[138] Kong Y, Hou X, Liu Z. et al. Cold-stress induced metabolomic and transcriptomic changes in leaves of three mango varieties with different cold tolerance. BMC Plant Biol. 2024;24:266 38600447 10.1186/s12870-024-04983-z PMC11005188

[139] Ma D, Sun D, Wang C. et al. Expression of flavonoid biosynthesis genes and accumulation of flavonoid in wheat leaves in response to drought stress. Plant Physiol Biochem. 2014;80:60–6 24727789 10.1016/j.plaphy.2014.03.024

[140] Han X, Li Y-H, Yao M-H. et al. Transcriptomics reveals the effect of short-term freezing on the signal transduction and metabolism of grapevine. Int J Mol Sci. 2023;24:3884 36835298 10.3390/ijms24043884 PMC9965549

[141] Ding C, Lei L, Yao L. et al. The involvements of calcium-dependent protein kinases and catechins in tea plant [*Camellia sinensis* (L.) O. Kuntze] cold responses. Plant Physiol Biochem. 2019;143:190–202 31518850 10.1016/j.plaphy.2019.09.005

[142] KhokharVoytas A, Shahbaz M, Maqsood MF. et al. Genetic modification strategies for enhancing plant resilience to abiotic stresses in the context of climate change. Funct Integr Genomics. 2023;23:283 37642792 10.1007/s10142-023-01202-0

[143] Alemu A, Åstrand J, Montesinos-López OA. et al. Genomic selection in plant breeding: key factors shaping two decades of progress. Mol Plant. 2024;17:552–78 38475993 10.1016/j.molp.2024.03.007

[144] Sheikh M, Iqra F, Ambreen H. et al. Integrating artificial intelligence and high-throughput phenotyping for crop improvement. J Integr Agric. 2024;23:1787–802

[145] Yoosefzadeh Najafabadi M, Hesami M, Eskandari M. Machine learning-assisted approaches in modernized plant breeding programs. Genes (Basel). 2023;14:777 37107535 10.3390/genes14040777 PMC10137951

[146] Drapal M, Enfissi EMA, Almeida J. et al. The potential of metabolomics in assessing global compositional changes resulting from the application of CRISPR/Cas9 technologies. Transgenic Res. 2023;32:265–78 37166587 10.1007/s11248-023-00347-9

[147] Nishihara M, Higuchi A, Watanabe A. et al. Application of the CRISPR/Cas9 system for modification of flower color in *Torenia fournieri*. BMC Plant Biol. 2018;18:331 30518324 10.1186/s12870-018-1539-3 PMC6280492

[148] Karlson CK, Mohd Noor SN, Khalid N. et al. CRISPRi-mediated down-regulation of the cinnamate-4-hydroxylase (C4H) gene enhances the flavonoid biosynthesis in *Nicotiana tabacum*. Biology. 2022;11:1127 36009753 10.3390/biology11081127 PMC9404795

[149] Wen D, Wu L, Wang M. et al. CRISPR/Cas9-mediated targeted mutagenesis of FtMYB45 promotes flavonoid biosynthesis in Tartary buckwheat (*Fagopyrum tataricum*). Front Plant Sci. 2022;13:879390 35646007 10.3389/fpls.2022.879390 PMC9133938

[150] Zhang P, Du H, Wang J. et al. Multiplex CRISPR/Cas9-mediated metabolic engineering increases soya bean isoflavone content and resistance to soya bean mosaic virus. Plant Biotechnol J. 2020;18:1384–95 31769589 10.1111/pbi.13302 PMC7206993

[151] Miranda S, Piazza S, Nuzzo F. et al. CRISPR/Cas9 genome-editing applied to *MdPGT1* in apple results in reduced foliar phloridzin without impacting plant growth. Plant J Cell Mol Biol. 2023;113:92–105 10.1111/tpj.16036 36401738

[152] Zhang X-H, Tee LY, Wang X-G. et al. Off-target effects in CRISPR/Cas9-mediated genome engineering. Mol Ther Nucleic Acids. 2015;4:e264 26575098 10.1038/mtna.2015.37 PMC4877446

[153] Hao Y . CRISPR-Cas system: off-target effects and its possible solutions. In: Alan W (ed.), International Conference on Biological Engineering and Medical Science. SPIE, US. 2024;12924:129242X

[154] Vakulskas CA, Dever DP, Rettig GR. et al. A high-fidelity Cas9 mutant delivered as a ribonucleoprotein complex enables efficient gene editing in human hematopoietic stem and progenitor cells. Nat Med. 2018;24:1216–24 30082871 10.1038/s41591-018-0137-0 PMC6107069

[155] Manghwar H, Li B, Ding X. et al. CRISPR/Cas systems in genome editing: methodologies and tools for sgRNA design, off-target evaluation, and strategies to mitigate off-target effects. Adv Sci. 2020;7:1902312 10.1002/advs.201902312 PMC7080517 32195078

[156] Marone D, Mastrangelo AM, Borrelli GM. et al. Specialized metabolites: physiological and biochemical role in stress resistance, strategies to improve their accumulation, and new applications in crop breeding and management. Plant Physiol Biochem. 2022;172:48–55 35030365 10.1016/j.plaphy.2021.12.037

[157] Ding T, Cao K, Fang W. et al. Evaluation of phenolic components (anthocyanins, flavanols, phenolic acids, and flavonols) and their antioxidant properties of peach fruits. Sci Hortic. 2020;268:109365

[158] Wang G, Xu M, Wang W. et al. Fortifying horticultural crops with essential amino acids: a review. Int J Mol Sci. 2017;18:1306 28629176 10.3390/ijms18061306 PMC5486127

[159] Wolter F, Schindele P, Puchta H. Plant breeding at the speed of light: the power of CRISPR/Cas to generate directed genetic diversity at multiple sites. BMC Plant Biol. 2019;19:176 31046670 10.1186/s12870-019-1775-1 PMC6498546

[160] Pramanik D, Shelake RM, Kim MJ. et al. CRISPR-mediated engineering across the central dogma in plant biology for basic research and crop improvement. Mol Plant. 2021;14:127–50 33152519 10.1016/j.molp.2020.11.002

[161] Zaidi S.S.-e.-A., Mahas A., Vanderschuren H. . et al. Engineering crops of the future: CRISPR approaches to develop climate-resilient and disease-resistant plants. Genome Biol. 2020;21:289 33256828 10.1186/s13059-020-02204-y PMC7702697

[162] Doggalli G, Monya D, Kumar MB. et al. Breeding techniques and approaches for developing abiotic stress-tolerant crop cultivars: a comprehensive review. Plant Cell Biotechnol Mol Biol. 2024;25:101–25

[163] Gulzar SS, Shabir HW, Hussain W. et al. Engineering cold stress tolerance in crop plants. Curr Genomics. 2011;12:30–43 21886453 10.2174/138920211794520178 PMC3129041

[164] Ahmad Y, Haakim Z, Iqbal J. et al. Technological innovations for abiotic stress resistance in horticultural crops. In: Fiaz S, Prakash CS (eds), OMICs-Based Techniques for Global Food Security. Hoboken, NJ, USA: Wiley-Blackwell, 2024, 233–44

[165] Nicholas R, Golan M, Fengde W. et al. Enhanced reproductive thermotolerance of the tomato *high pigment 2* mutant is associated with increased accumulation of flavonols in pollen. Front Plant Sci. 2021;12:672368 34093629 10.3389/fpls.2021.672368 PMC8171326

[166] Le Gall G, DuPont MS, Mellon FA. et al. Characterization and content of flavonoid glycosides in genetically modified tomato (*Lycopersicon esculentum*) fruits. J Agric Food Chem. 2003;51:2438–46 12696918 10.1021/jf025995e

[167] Xiao F, Yang Z, Han W. et al. Effects of day and night temperature on photosynthesis, antioxidant enzyme activities, and endogenous hormones in tomato leaves during the flowering stage. J Hortic Sci Biotechnol. 2018;93:306–15

[168] Castillejo C, Waurich V, Wagner H. et al. Allelic variation of *MYB10* is the major force controlling natural variation in skin and flesh color in strawberry (*Fragaria* spp.). Fruit Plant Cell. 2020;32:3723–49 33004617 10.1105/tpc.20.00474 PMC7721342

[169] Wang H, Zhang H, Yang Y. et al. The control of red colour by a family of MYB transcription factors in octoploid strawberry (*Fragaria* × *ananassa*) fruits. Plant Biotechnol J. 2019;18:1169–84 31647169 10.1111/pbi.13282 PMC7152614

[170] Shirzad H, Alirezalu A, Alirezalu K. et al. Effect of *Aloysia citrodora* essential oil on biochemicals, antioxidant characteristics, and shelf life of strawberry fruit during storage. Meta. 2021;11:256 10.3390/metabo11050256 PMC8143293 33919369

[171] Król-Grzymała A, Amarowicz R. Phenolic compounds of soybean seeds from two European countries and their antioxidant properties. Molecules. 2020;25:2075 32365546 10.3390/molecules25092075 PMC7249021

[172] Tan S, Zhu Y, Wang Y. et al. Refrigerated storage stimulates isoflavone and γ-aminobutyric acid accumulation in germinated soybeans. Plant Physiol Biochem. 2024;210:108667 38678946 10.1016/j.plaphy.2024.108667

[173] Ahmad MZ, Li P, Wang J. et al. Isoflavone malonyltransferases GmIMaT1 and GmIMaT3 differently modify isoflavone glucosides in soybean (*Glycine max*) under various stresses. Front Plant Sci. 2017;8:735 28559900 10.3389/fpls.2017.00735 PMC5433297

[174] Jimenes IM, Silva SR, Tezotto-Uliana JV. et al. Fruit quality attributes of low chilling requirement ‘Snowchaser’ blueberry cultivated in Brazil. Rev Bras Frutic. 2018;40:e-473

[175] Ge Y, Tang Q, Li C. et al. Acibenzolar-S-methyl treatment enhances antioxidant ability and phenylpropanoid pathway of blueberries during low temperature storage. LWT. 2019;110:48–53

[176] Chen W, Minna Z, Limin W. et al. Qualitative and quantitative analysis of phenolic compounds in blueberries and protective effects on hydrogen peroxide-induced cell injury. J Sep Sci. 2021;44:2837–55 33939882 10.1002/jssc.202001264

[177] Dai H, Li B, Song Z. et al. Anthocyanin treatment delays the senescence of blueberry fruit by regulating abscisic acid and anthocyanin synthesis processes. Food Qual Saf. 2023;8:fyad053

[178] Balentine DA, Dwyer JT, Erdman JW. et al. Recommendations on reporting requirements for flavonoids in research. Am J Clin Nutr. 2015;101:1113–25 25854881 10.3945/ajcn.113.071274

[179] Zhang S, Wang R, Zhao Y. et al. Biotransformation of myricetin: a novel metabolic pathway to produce aminated products in mice. Mol Nutr Food Res. 2019;63:e1900203 31087612 10.1002/mnfr.201900203

[180] Wahnou H, Limami Y, Oudghiri M. Flavonoids and flavonoid-based nanoparticles for osteoarthritis and rheumatoid arthritis management. Biochemist. 2024;4:38–61

[181] Panche AN, Diwan AD, Chandra SR. Flavonoids: an overview. J Nutr Sci. 2016;5:e47 28620474 10.1017/jns.2016.41 PMC5465813

[182] Wahnou H, Liagre B, Sol V. et al. Polyphenol-based nanoparticles: a promising frontier for enhanced colorectal cancer treatment. Cancers. 2023;15:3826 37568642 10.3390/cancers15153826 PMC10416951

[183] Ongtanasup T, Kamdenlek P, Manaspon C. et al. Green-synthesized silver nanoparticles from *Zingiber officinale* extract: antioxidant potential, biocompatibility, anti-LOX properties, and in silico analysis. BMC Complement Med Ther. 2024;24:84 38350963 10.1186/s12906-024-04381-w PMC10863109

[184] Wang L, Chen M, Lam P-Y. et al. Multifaceted roles of flavonoids mediating plant-microbe interactions. Microbiome. 2022;10:233 36527160 10.1186/s40168-022-01420-x PMC9756786

